# Comparison of Mechanical Property Simulations with Results of Limited Flexural Tests of Different Multi-Layer Carbon Fiber-Reinforced Polymer Composites

**DOI:** 10.3390/polym16111588

**Published:** 2024-06-03

**Authors:** Ronald Bastovansky, Lukas Smetanka, Robert Kohar, Rajesh Kumar Mishra, Michal Petru

**Affiliations:** 1 Department of Design and Machine Elements, Faculty of Mechanical Engineering, University of Žilina, Univerzitná 8215/1, 010 26 Žilina, Slovakia; ronald.bastovansky@fstroj.uniza.sk (R.B.); lukas.smetanka@fstroj.uniza.sk (L.S.); 2 Department of Material Science and Manufacturing Technology, Faculty of Engineering, Czech University of Life Sciences Prague, Kamycka 129, 165 00 Prague, Czech Republic; mishrar@tf.czu.cz; 3 Department of Machine Parts and Mechanism, Faculty of Mechanical Engineering, Technical University of Liberec, Studentská 1402/2, 461 17 Liberec, Czech Republic; michal.petru@tul.cz

**Keywords:** carbon fiber-reinforced composite, flexural test, elastic mechanical properties, FEM, simulation, analysis, Digimat software, MSC Marc Mentat software

## Abstract

This article is focused on the experimental study of flexural properties in different multi-layer carbon fiber-reinforced polymer (CFRP) composites and correlations with the results of finite element method (FEM) simulations of mechanical properties. The comparison of the results shows the possibility of reducing the number of experimental specimens for testing. The experimental study of flexural properties for four types of carbon fiber-reinforced polymer matrix composites with twill weaves (2 × 2) was carried out. As input materials, pre-impregnated carbon laminate GG 204 T and GG 630 T (prepreg) and two types of carbon fiber fabrics (GG 285 T and GG 300 T (fabric)) were used. Multi-layer samples were manufactured from two types of prepregs and two types of fabrics, which were hand-impregnated during sample preparation. The layers were stacked using same orientation. All specimens for flexural test were cut with the longer side in the weft direction. Pre-impregnated carbon laminates were further impregnated with resin DT 121H. Carbon fabrics were hand-impregnated with epoxy matrix LG 120 and hardener HG 700. To fulfill the aim of this research, finite element method (FEM)-based simulations of mechanical properties were performed. The FEM simulations and analysis were conducted in Hexagon’s MSC Marc Mentat 2022.3 and Digimat 2022.4 software. This paper presents the results of actual experimental bending tests and the results of simulations of bending tests for different composite materials (mentioned previously). We created material models for simulations based on two methods—MF (Mean Field) and FE (Finite Element), and the comparative results show better agreement with the MF model. The composites (GG 285 T and GG 300 T) showed better flexural results than composites made from pre-impregnated carbon laminates (GG 204 T and GG 630 T). The difference in results for the hand-impregnated laminates was about 15% higher than for prepregs, but this is still within an acceptable tolerance as per the reported literature. The highest percentage difference of 14.25% between the simulation and the real experiment was found for the software tool Digimat FE 2022.4—GG 630 T composite. The lowest difference of 0.5% was found for the software tool Digimat MF 2022.4—GG 204 T composite. By comparing the results of the software tools with the results of the experimental measurements, it was found that the Digimat MF 2022.4 tool is closer to the results of the experimental measurements than the Digimat FE 2022.4 tool.

## 1. Introduction

Composite materials have a wide range of applications in the aerospace, automotive, civil engineering, and naval construction industries and in structures for the manufacture of wind energy and sports goods, as well as special applications where low weight and high strength are required [1,2,3,4,5,6,7,8,9]. Polymer composite materials show high resistance to corrosion and allow for creating components with a complex shape [1,2,3,4,5,6,7,8,9,10]. All composite materials are generally composed of two phases: the reinforcement (fibers or particles) and the matrix. The properties of the final composite material depend on the properties of the reinforcement and the matrix [1,2,3,4,5]. Basically, the composite materials are divided according to their structural components, structural design or matrix material. The functions of the matrix bind the reinforcement together, mechanically support the reinforcement, transfer the load to the reinforcement, and protect the reinforcement from surface damage due to abrasion or chemical attacks. Reinforcement is the phase enveloped by the matrix. Reinforcement is the stronger and stiffer integral component of the composite that usually bears the majority of the applied stresses upon mechanical loading [2]. This paper is focused on carbon fiber-reinforced polymer (CFRP) composites. Carbon fibers are the stiffest and strongest reinforcing fibers generally used for polymer composites. They are the most used after glass or other high performance industrial fibers. Carbon fibers are made of pure carbon in the form of graphite, which has a relatively lower density and a negative coefficient of longitudinal thermal expansion. They are generally used together with epoxy. Carbon fibers are produced from polyacrylonitrile (PAN) or the pitch methods [2,11,12]. For the manufacture of composite materials, woven carbon fabric is widely used. Basically plain, twill or satin weave patterns are used in composite materials. The woven fabrics are attractive for their low fabrication cost and easy hand layup for structural applications. [1,2,13,14]. Fabric design can influence the mechanical properties required for the composite material application. As mentioned in the literature [14], by changing the weave pattern, fiber type, yarn type, and yarn fineness, the required mechanical properties can be optimized. The diversity of fabric designs is interesting.

The selection of suitable input materials and the design of the composite structure for a particular application can be challenging in the initial design process. Currently, this problem can be partially solved with the help of CAD systems. FEM simulations of models designed from selected input materials can be very helpful in this respect. However, several input data are required for FEM simulations of the required mechanical properties in carbon fiber-reinforced polymer (CFRP) materials [4,9,15]. These input data must be obtained using experimental measurements of mechanical properties. Data obtained from flexural test and data from structural analysis of real composite samples are used in FEM simulation. The modeling and prediction of the mechanical properties of woven laminates using the FEM technique are described in the literature [4,9,13,14,15,16,17]. According to the literature [17], modeling elastic properties usually requires the characterization of five independent elastic constants, which represent Young’s modulus in the fiber direction, Young’s modulus perpendicular to the fiber direction, the in-plane shear modulus, the in-plane Poisson’s ratio, the out-of-plane shear modulus, and the out-of-plane Poisson’s ratio. The FEM simulations and analysis were conducted in Hexagon’s MSC Marc Mentat and Digimat software. The FEM method is not the only one that can be used to simulate the behavior of composite materials. Yuanyuan Wang et al. [18] successfully used the decomposition generalized finite difference method for stress analysis in three-dimensional composite materials. The GFDM method has emerged as a robust meshless method for various engineering applications. This method uses Taylor series expansions and moving least-squares approximation to derive explicit formulae for the required partial derivatives of unknown variables. Kabir et al. [19] and Heydarpour et al. [20] used the Bézier-based multi-step method for solving the composite material problems. As an alternate numerical solution technique for the analysis of composite structures, the differential quadrature method (DQM) can be used [21]. The flexural properties of polymer-reinforced composites are determined by norm ASTM D7264 [22] or by EN ISO 14125:1998 [23].

In this case, a fabric with a twill weave was used. It is known that a twill weave has a looser interlacement, and the weave is characterized by a diagonal line [14]. A twill weave is more flexible than a plain weave and has better grip while maintaining more fabric stability than satin weave. A twill weave also wets out better than a plain weave [2,4,14]. Woven fabrics are generally used together with epoxy resin (matrix). Carbon fabrics with twill pattern 2/2 was used in this study. They were hand-impregnated with epoxy matrix LG 120 and hardener HG 700 [24]. The input materials for manufacture of composites are pre-impregnated carbon laminates. Two types of prepregs (from company G. Angeloni) with the same pattern (2/2 twill) were used as experimental materials [21]. Pre-impregnation was performed using the resin DT 121H [25]. The detailed preparation of the composite material is described in main text of the article. The input data used for modeling in Digimat software were from the manufacturer’s datasheets, but the verification was performed in line with the method of Xian et al. for glass fiber [26]. It is also possible to verify the mechanical properties of epoxy resin, as reported by Wu Jingyu et al. [27]. More information about composite manufacturing is possible to study in [1,28,29,30,31,32].

This work is focused on experimentally studying flexural properties for different multi-layer CFRP composites and their correlation with the results of FEM simulations of mechanical properties. A comparison of the results shows the possibility of reducing the number of experimental specimens. The motivation was to develop the model while taking into account prepregs and the type of fabric material, which is not so common, and little has been reported in this regard.

## 2. Materials and Methods

### 2.1. Input Materials

For the experimental study of CFRP composite mechanical behavior, four types of input materials were selected. The first two are pre-impregnated carbon laminate GG 204T and GG 630T (prepregs). The next two are carbon fiber-based fabrics GG 285T and GG300T (fabrics), which were hand-impregnated. Basic material properties according to the manufacturers are presented in Table 1.

Pre-impregnated carbon laminates were further impregnated with resin DT 121H. For the hand-impregnation of carbon fabrics, epoxy matrix LG 120 and hardener HG 700 was used. The mechanical and thermal properties of resin 120 and hardener HG 700 are defined according to the technical sheet of GRM System company [24], presented in Table 2, Table 3 and Table 4.

**Figure 1:**
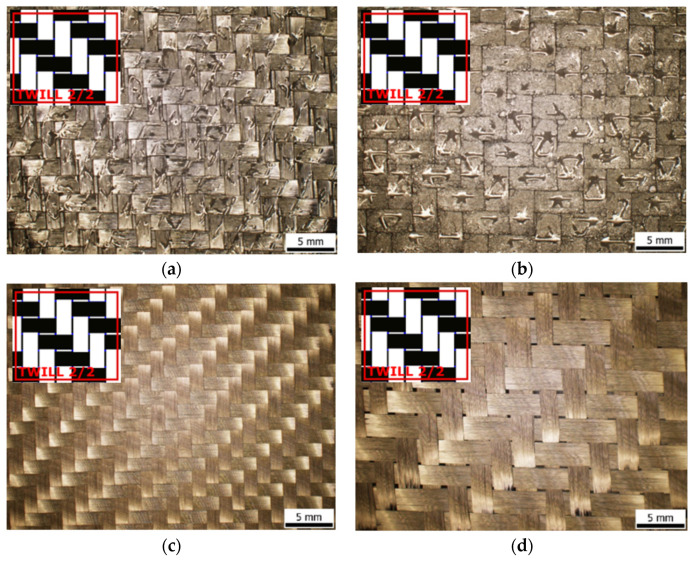
Structure of presented input materials: (**a**) prepreg GG 204 T; (**b**) prepreg GG 630 T; (**c**) fabric GG 285 T, (**d**) fabric GG 300 T.

The company Delta Preg [28] manufactured the pre-impregnated carbon laminate (prepreg). The company G. Angeloni [25] manufactured the carbon fabrics. All fabric materials were developed with a twill weave (2 × 2). Macrostructures of the experimental materials and scheme of weaving are presented on Figure 1. Photos were observed by an optical Olympus stereomicroscope.

From these presented input materials, samples of CFRP composites were prepared. The detailed preparation method is mentioned in the following subsections.

### 2.2. Preparation of CFRP Composite Sample from GG 204 T and GG 630 T

**Figure 2:**
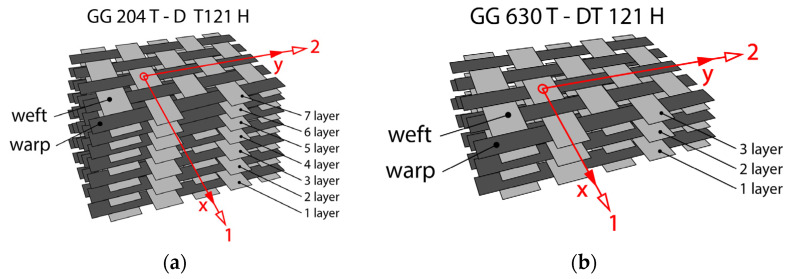
Schematic image of composite samples from prepregs: hand lay up of (**a**) seven layers of prepreg GG 204 T-DT 121H; and (**b**) three layers of prepreg GG 630 T-DT 121H.

Multi-layer samples were manufactured from pre-impregnated carbon laminate. Seven layers were stacked from prepreg GG 204 T (see Figure 2a), and from prepreg GG 630 T, three layers were stacked by hand lay-up (see Figure 2b). The method of layering was influenced by different thicknesses of the prepregs, so it was necessary to ensure a standardized thickness of 2 mm for all the samples for the bending test. The layers were stacked with the same orientation. Attention was paid to the fact that all specimens could be cut with the longer side in the weft direction x (see Figure 2).

**Figure 3:**
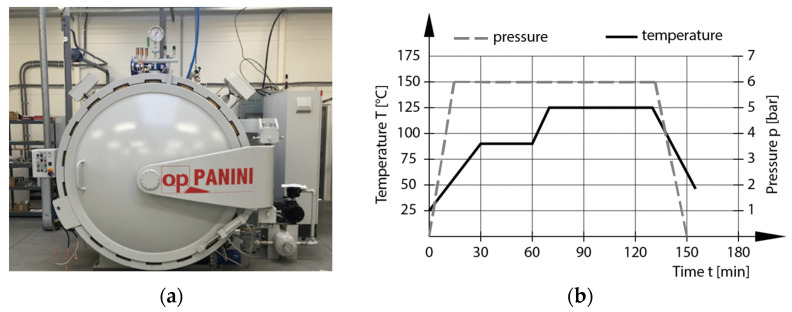
Autoclave Panini in R&D mold machining company (**a**). A Curing process of GG 204 T and GG 630 T composite materials (**b**).

The stacked layers of prepregs GG 204 T and GG 630 T were placed on steel plates. The separation of the prepreg from the plate was performed with Partall Paste#2 wax. Steel plates with stacked layers of prepreg were placed in vacuum bags. Then, the bags were vacuumed to 0.01 MPa and placed in a Panini autoclave (see Figure 3a). The composite samples were cured at the required temperature and pressure conditions for the prescribed time, according to the manufacturer’s recommendation. The autoclave curing process is shown in Figure 3b. The general characteristics for the autoclave Panini from R&D mold machining company are as follows: internal dimensions—diameter: 1500 mm, length: 3000 mm; working pressure—from 0.5 to 11.5 bar; working temperature—up to 280 °C, 22 thermocouples; and thermal gradient—from 0.5 to 10 °C [24].

### 2.3. Preparation of CFRP Composite Samples from GG 285 T and GG 300 T

**Figure 4:**
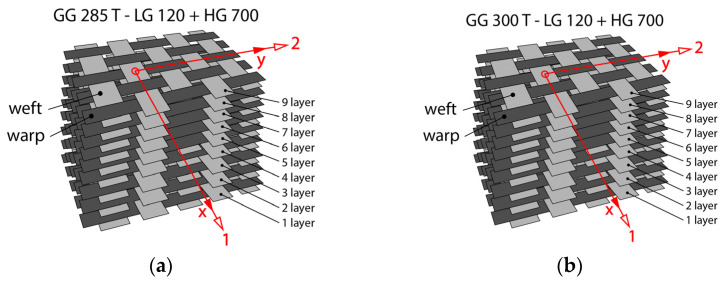
Composite samples—schematic image of hand lay up of (**a**) nine layers of fabric GG 285 T; and (**b**) nine layers of fabric GG 300 T.

Composite samples from carbon fiber-based fabrics were made for GG 285 T and GG 300 T. For each sample, nine layers were used (see Figure 4). The system with LG 120 epoxy resin and HG 700 hardener was used for the manual lamination of the nine stacked layers. Their properties are listed in Table 2. The cut layers were manually impregnated one by one on the stainless-steel base plate. After impregnation and laying all layers, a second stainless-steel plate was placed on the top layer. The stainless-steel plates were separated by Partall Paste#2 wax before placing the impregnated fabric layers. The plates were loaded with flexible clamps. The samples were cured at a temperature of 24 °C for 24 h under atmospheric pressure.

### 2.4. Preparation of Specimen for Flexural Testing

**Figure 5:**
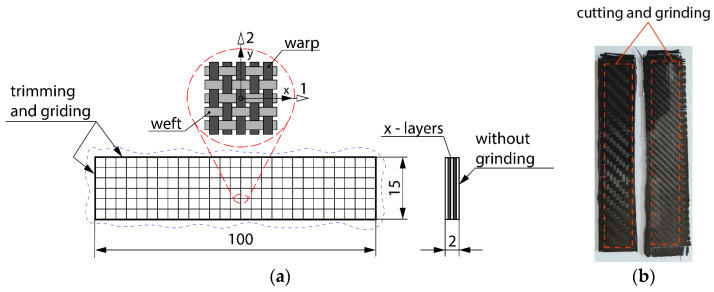
Specimen for flexural test according to standard ISO 14 125: (**a**) geometry (dimension in millimeters), determination of specimen surface treatments, and specimen orientation in direction of weft; (**b**) samples of composite for cutting and grinding of specimens.

After curing processes, all samples of composite laminates were cut in line with the specimen with required dimensions for flexural tests according to standards (see Figure 5). They were cut using a diamond circular blade. The specimens with defined shapes and dimensions were manufactured in accordance with standard ISO 14 125: Fiber-reinforced plastic composites—Determination of flexural properties [19].

**Figure 6:**
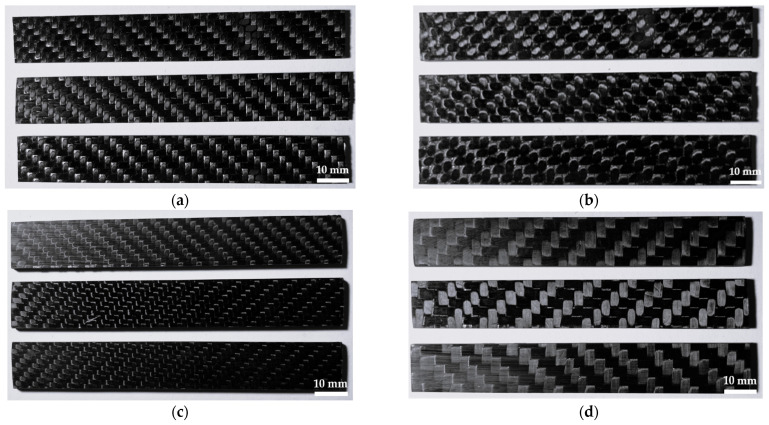
Multi-layer CFRP composite specimens for flexural test: (**a**) composite GG 204 T-DT 121H; (**b**) composite GG 630 T-DT 121H; (**c**) composite GG 285 T-LG 120 + HG 700, (**d**) composite GG 300 T-LG 120 + HG 700.

The dimensions for each specimen for flexural tests are shown in Table 5. Specimens were cut with their longer side oriented in direction 1 (weft), as was mentioned earlier. After cutting, surfaces around the perimeter were ground. Both surfaces of the specimens with dimensions of 100 × 15 mm remained in their original state. Three specimens of each composite sample were prepared for experimental flexural tests (see Figure 6).

### 2.5. Flexural Test Method

The flexural test is essentially a three-point bending test. Three-point bending was performed on three samples for each type of composite according to standard ISO 14 125. LabTest 3.20 machines with a maximum load of 3 kN were used for this test. The test was performed in a laboratory at the University of Žilina, Faculty of Mechanical Engineering, Department of Design and Mechanical Elements.

**Figure 7:**
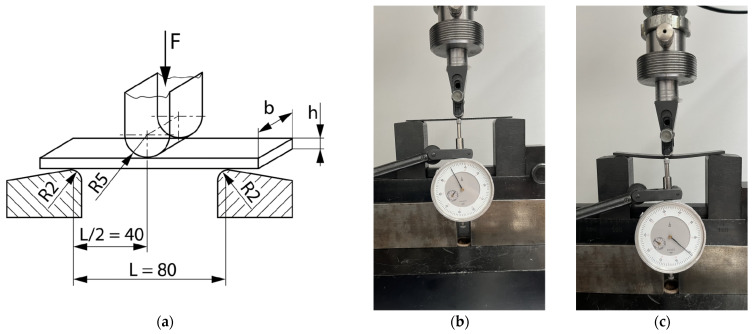
Flexural test: (**a**) Schematic view of sample storage on supports, dimensions; (**b**) real view of test—unloaded specimen; (**c**) real view of test—specimen loaded.

The method of placing the specimens on the supports, the dimensions and shape of the supports, and the load mandrel are shown in Figure 7. The distance between the supports was determined according to the standard at a distance of L = 80 mm, which represents the ratio of L/h = 40 mm. The flexural tests were performed under laboratory conditions (temperature of 22 °C, humidity 58%).

The specimens were loaded with a mandrel located in the middle between the L/2 supports. The speed of the load mandrel was 2 mm/min. For the purposes of this experiment, the specimens were loaded only in the elastic region. Note: the samples were loaded until the moment of the first audible cracking sounds, and then the test was stopped. Deformation deflection was recorded from a dial gauge, which was placed on the underside of the loading mandrel. The accuracy of the dial gauge was 0.01 mm. The force–deformation curve was recorded during the test. After the test, each specimen was loaded again. The force–deformation load curves were compared. In this way, we determined if the specimen had failed and if the first loading had not exceeded the elastic area.

## 3. FEM Analysis

### 3.1. Software Used for Modeling of Composite Material

The simulation and modeling for the simulation were performed in Hexagon’s MSC Marc Mentat 2022.3 software. For the analysis of material, we also used Digimat 2022.4 software.

MSC Marc Mentat 2022.3 software is a nonlinear finite elements analysis software used to simulate the behavior of complex materials and interaction under large deformations and strains. MSC Marc Mentat 2022.3 software was used to perform finite element analyses of structures, accounting for all nonlinearities, in one, two, and three dimensions. MSC Marc Mentat 2022.3 software can be used to run various types of mechanical simulations [28].

For the prediction of the nonlinear micromechanical behavior of complex multiphase composite materials and structures, engineers and material specialists use the software Digimat.

The Digimat platform offers three different categories of products [28]:

1. Tools: For the analysis of nonlinear multi-scale composite materials (MF, FE, MX–performance; MAP, CAE–manufacturing).
2. Solutions: Use of Digimat technology in a fully integrated GUI controlled environment for specific tasks (RP, VA, AM).
3. Expertise: Consists of a User’s Manual, including a manual support center and a service center.

**Figure 8:**
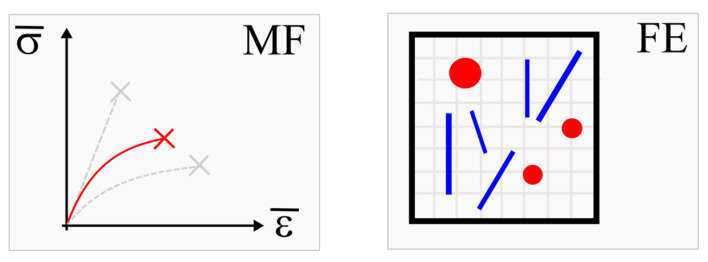
Digimat MF 2022.4 (Mean Field) homogenization software tool and Digimat FE 2022.4 (Finite Element) homogenization software tool.

For more information about Digimat platform categories, please see [28]. For the simulations presented in this work, a Digimat MF (Mean Field) homogenization tool and an FE (Finite Element) homogenization tool were used (see Figure 8).

Digimat MF [26] is the mean-field homogenization tool used to quickly compute the macroscopic performance of composite materials from their per-phase properties and microstructure definition. Digimat MF aims at the realistic prediction of the nonlinear constitutive properties of multi-phase materials, taking into account temperature and strain rate dependencies. The composite morphology, including filler content, length, aspect ratio, and orientation, fully impacts the resulting composite properties. Digimat Mean Field homogenization technology is especially well suited to describe fiber-reinforced composites.

Digimat-FE [26] generates realistic representative volume elements (RVEs) for a large variety of material microstructures and can also rely on external geometric microstructure descriptions such as micro-CT-scan or molecular dynamic results.

Based on material input and the microstructure definition, a finite element model is built and run. Various solvers are accessible to perform simulations, including an embedded FEA solver, a fast Fourier transform solver, and external solvers. The results of the FE analysis are post-processed in the sense of probabilistic distribution functions that give detailed insight into the RVEs. Mean homogenized values are computed and can be used in subsequent FE analysis on the structural part level [26].

### 3.2. Determination of Material Properties in Digimat MF and FE Software Tools

The fabric microstructure module was used to define material models. When using the Digimat MF 2022.4 software tool, the steps are:

1. Definition of the matrix—in this step, the properties of the material that forms it are assigned to the matrix.
2. Definition of yarn phase—fiber density, fiber diameter, and yarn cross-section.
3. Definition of weaving—yarn crimp, number of warp and weft yarns, and their spacing.

The input data that were used in the model simulations of individual material prepreg and fabrics of composites are shown in Table 6.

The properties of the entire composite material were calculated in the program after entering all the necessary properties of the matrix, yarn phase and fabric weaving parameters.

The subsequent definition of the input data of individual composite structures in the Digimat FE software tool is similar to that for the Digimat MF 2022.4 software tool.

**Figure 9:**
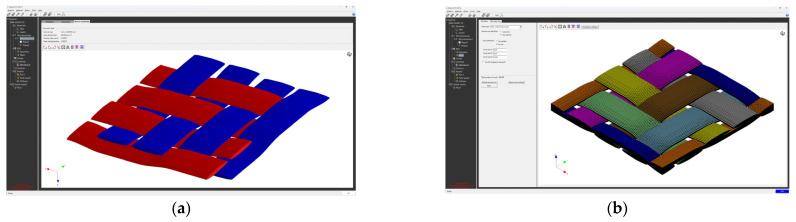
Model visualization for GG 204 T material: (**a**) visualization of the woven fabric structure; (**b**) visualization of the mesh of the composite model.

**Figure 10:**
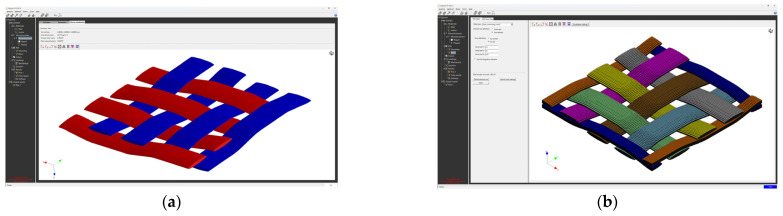
Model visualization for GG 630 T material: (**a**) visualization of the woven fabric structure; (**b**) visualization of the mesh of the composite model.

**Figure 11:**
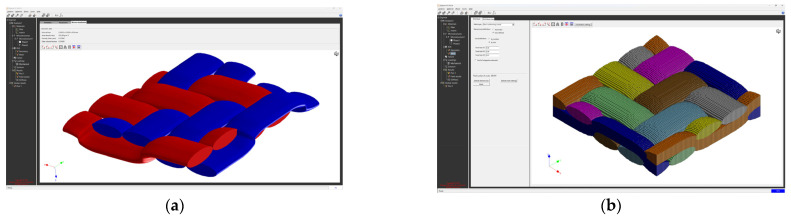
Model visualization for GG 285 T material: (**a**) visualization of the woven fabric structure; (**b**) visualization of the mesh of the composite model.

**Figure 12:**
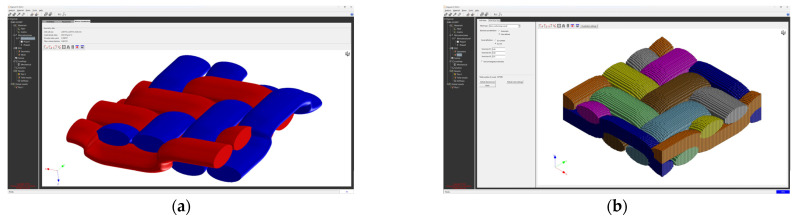
Model visualization for GG 300 T material: (**a**) visualization of the woven fabric structure; (**b**) visualization of the mesh of the composite model.

The creation of the FEM mesh followed the entry of all input parameters. After defining all parameters of the material model, its material characteristics were subsequently calculated using FEM analysis. The visualization of the woven fabric structure and meshing visualization of the virtual composite models are shown in the following (Figure 9, Figure 10, Figure 11 and Figure 12).

### 3.3. FEM Analysis in MSC Marc Mentat

**Figure 13:**
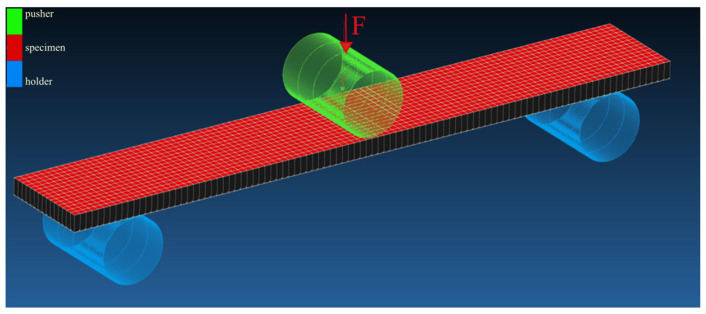
Visualization of experimental specimen arrangement in MSC Marc Mentat 2022.3 software, mesh element is 2 mm.

The FEM simulations were performed in MSC Marc Mentat 2022.3 software from Hexagon. The simulations were conducted on the virtual test specimens (see Figure 13). The virtual specimen’s dimensions were calculated as average values of real specimens 1, 2, and 3. The dimensions of the real samples are shown in Table 5, and for virtual model specimens, they are shown in Table 6.

The modeled supports (Figure 13) for the virtual specimen have the same dimensions as in the real flexural test, and they meet the requirements stated in standard ISO 14125 [23]. They are defined as geometric surfaces. The contact between the supports and the virtual specimen is defined as contact interaction of the touch type, with a contact friction coefficient of value 0.1. A contact interaction of the touch type is also defined between the loading mandrel and the virtual sample, with a contact friction coefficient of value 0.1. At the same time, the loading mandrel acts on the virtual specimen with the force F (Figure 13), which was defined as the average value of the forces (Table 7, above) that were measured at the deflection of 4 mm of real specimens.

## 4. Results and Discussion

### 4.1. Results of Flexural Test

Dependence curves of flexural stress σ [MPa] on flexural strain ε [%] were obtained by experimental measurement using a three-point bending test.

**Figure 14:**
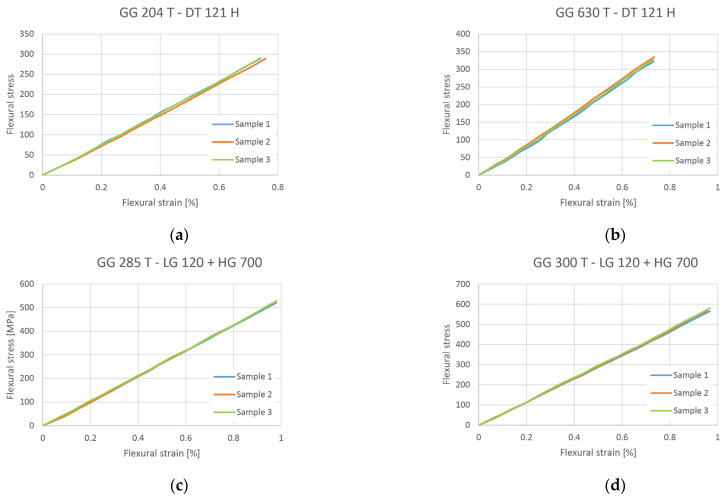
Dependence curve for flexural stress–flexural strain: (**a**) composite GG 204 T; (**b**) composite GG 630 T; (**c**) composite GG 285 T and (**d**) composite GG 300 T.

The flexural test was performed for all designed composites. The test was performed only in the elastic region. The speed of loading the specimen was 2 mm/min. The recorded loading dependence curves from these tests are presented in Figure 14.

The average flexural modulus *E_f_* was obtained using Equation (1) and the calculated values are presented in Table 8. Also, the flexural modulus for each tested composite is presented.

*E_f_* = (*σ*_*f*2_ − *σ*_*f*1_)/(*Ɛ*_*f*2_ − *Ɛ*_*f*1_), (1)

In the equation, according to standard ISO 14 125, *σ_f_*_1_ is the flexural stress in [MPa], measured at deflection s_1_ (deflection *s*_1_ corresponding to the given values of the flexural strain *Ɛ_f_*_1_ = 0.0005); *σ_f_*_2_ is the flexural stress in [MPa], measured at deflection s_2_ (deflection *s*_2_ corresponding to the given values of the flexural strain *Ɛ_f1_* = 0.0025).

*s_i_* = *Ɛ_fi_*
*L*^2^/(6 h) (*i* = 1 or 2), (2)

where *L* is distance between supports in [mm]; *h* is thickness of specimen in [mm].

**Figure 15:**
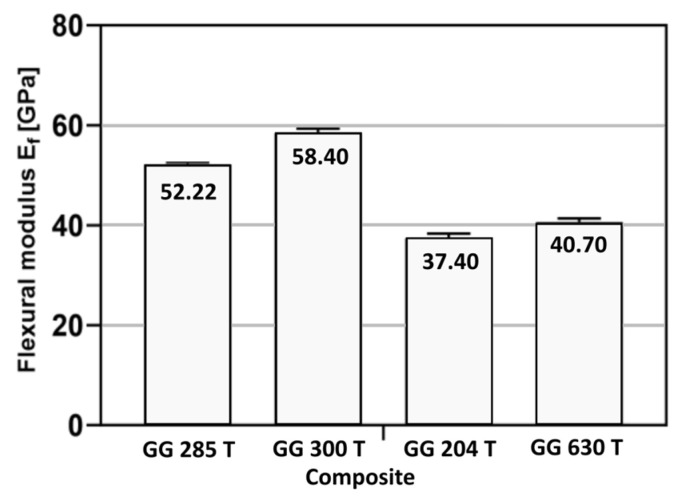
Value of average flexural modulus of elasticity for each composite.

Graphically presented values of the average flexural modulus for each composite material are presented on Figure 15.

The composites GG 285 T and GG 300 T showed better results than composites made from pre-impregnated carbon laminates (GG 204 T and GG 630 T).

### 4.2. Results from Digimat FE and MF Software Tools

**Figure 16:**
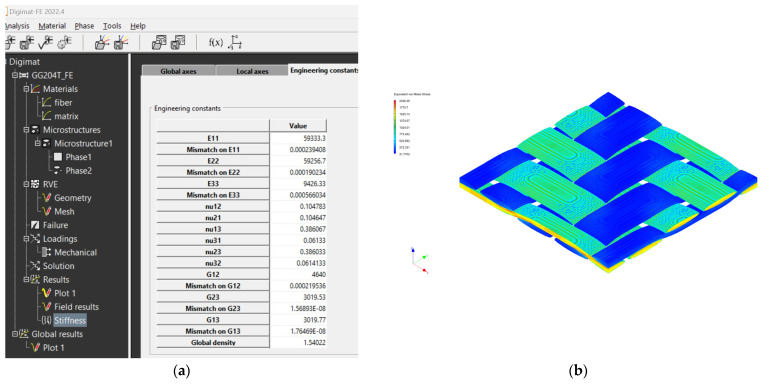
Output data from Digimat FE tool for composite GG 204 T: (**a**) calculated values of properties; (**b**) visualization of stresses during FEM analysis.

**Figure 17:**
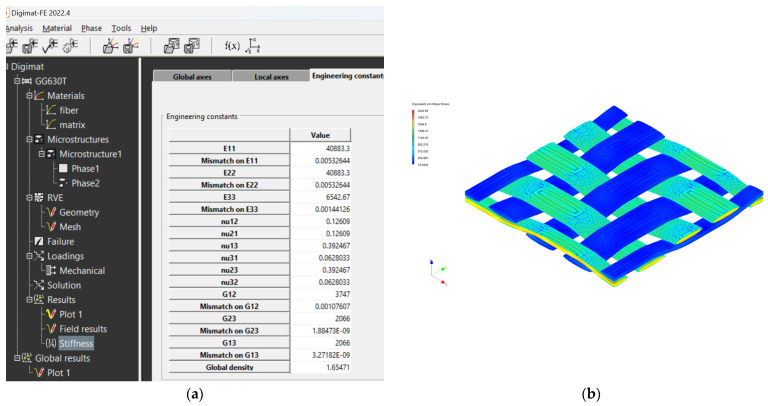
Output data from Digimat FE tool for composite GG 630 T: (**a**) calculated values of properties; (**b**) visualization of stresses during FEM analysis.

**Figure 18:**
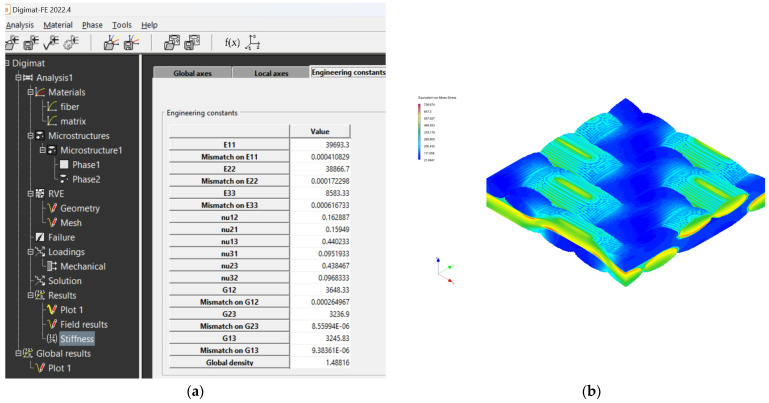
Output data from Digimat FE tool for composite GG 285 T: (**a**) calculated values of properties; (**b**) visualization of stresses during FEM analysis.

**Figure 19:**
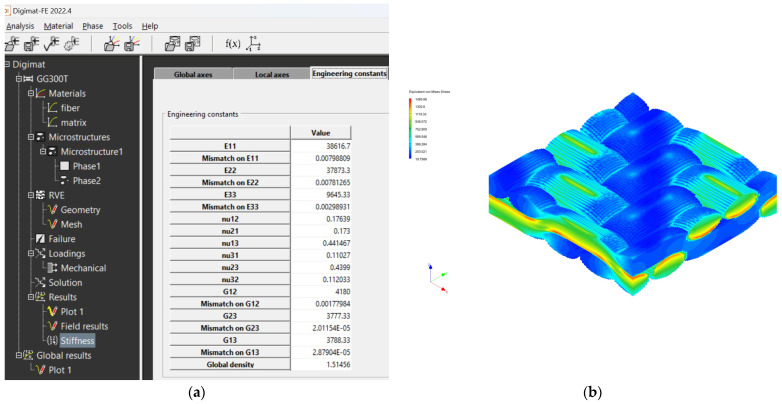
Output data from Digimat FE tool for composite GG 300 T: (**a**) calculated values of properties; (**b**) visualization of stresses during FEM analysis.

The mechanical properties of composite materials were investigated by simulation analysis. They were investigated by material simulation modeling according to output data from Digimat tools. These simulations are presented for each composite material: see Figure 16 for the GG 204 T composite, Figure 17 for the GG 630 composite, Figure 18 for the GG 285 T composite, and Figure 19 for the GG 300T composite.

**Figure 20:**
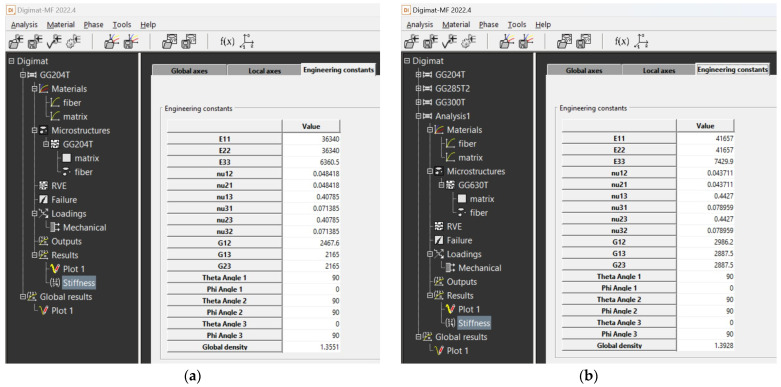
Output data from Digimat MF tool for composite: (**a**) calculated values for composite GG 204 T; (**b**) calculated values for composite GG 630 T.

**Figure 21:**
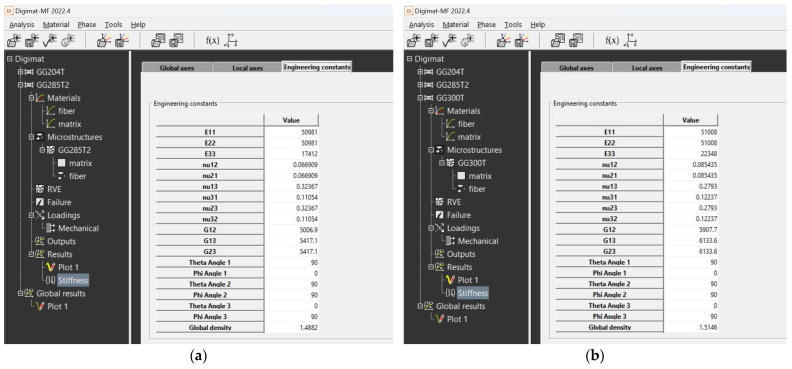
Output data from Digimat MF tool for composite: (**a**) calculated values for composite GG 258 T; (**b**) calculated values for composite GG 300 T.

The calculation of material properties in the Digimat MF tool was performed numerically; therefore, the resulting output is in the form of tables in which the calculated material properties can be seen (Figure 20 and Figure 21).

### 4.3. Results from MSC Marc Mentat Software

**Figure 22:**
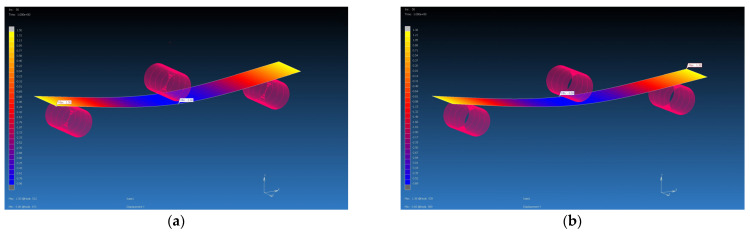
Flexural test simulation, deflection visualization from MSC Marc Mentat for GG 204 T composite, F = 142 N: (**a**) input data from Digimat MF, deflection is 3.98 mm; (**b**) input data from Digimat FE, deflection is 3.69 mm.

**Figure 23:**
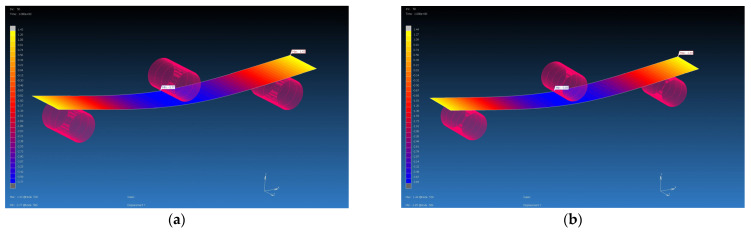
Flexural test simulation, deflection visualization from MSC Marc Mentat for GG 630 T composite, F = 156 N: (**a**) input data from Digimat MF, deflection is 3.77 mm; (**b**) input data from Digimat FE, deflection is 3.85 mm.

**Figure 24:**
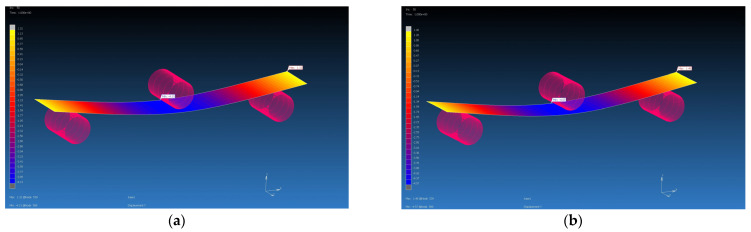
Flexural test simulation, deflection visualization from MSC Marc Mentat for GG 285 T composite, F = 445 N: (**a**) input data from Digimat MF, deflection is 4.13 mm; (**b**) input data from Digimat FE, deflection is 4.57 mm.

**Figure 25:**
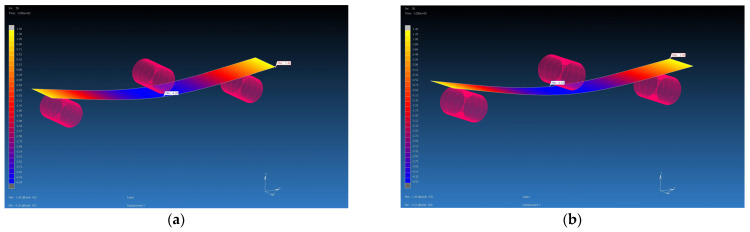
Flexural test simulation, deflection visualization from MSC Marc Mentat for GG 300 T composite, F = 467 N: (**a**) input data from Digimat MF, deflection is 4.29 mm; (**b**) input data from Digimat FE, deflection is 4.53 mm.

The calculated material properties in the Digimat program using the MF and FE methods were subsequently used as input parameters for the FEM simulation. The FEM simulation was carried out in the MSC Marc Mentat program. Using the FEM simulation, the values of the deflection of the virtual specimen, which was loaded with the calculated average value of the forces on the real specimen, were obtained. They are shown in Table 7. The deflections from the FEM simulations were compared with the value of the deflection of the real samples, which was 4 mm. Additionally, percentage evaluations of differences were performed (see Table 9, Figure 22, Figure 23, Figure 24 and Figure 25). The flexural modulus from FEM simulations was compared with the value of the flexural modulus of the real samples (Table 10).

The deviation between the FEM simulation results in MSC Marc Mentat deflection and the flexural modulus using the material models obtained using Digimat MF and FE tools and the measured actual deflection values and flexural modulus values of real samples was expressed as a percentage difference (see Table 10). In this research, only deflection in real samples was compared with simulations. The ultimate load-carrying capacity also depends on the flexural rigidity, which is, of course, determined by the deflection. A higher deflection indicates lower rigidity and possibly lower load-bearing capacity.

## 5. Conclusions

This article presents the results of real experimental bending tests and the results of simulations of bending tests for different multi-layer composite materials made of carbon prepregs (GG 204 T and GG 630 T (pre-impregnated with resin DT 121H)) and carbon fiber fabrics (GG 285 T and GG 300 T (impregnated with resin LG 120 and hardener HG 700)). Even the curing method was different for both series of samples. Composites GG 285 T and GG 300 T showed better flexural results than composites made from pre-impregnated carbon laminates GG 204 T and GG 630 T. The difference in results for the hand-impregnated laminates was about 15% higher than for prepregs, but this is still within an acceptable tolerance as per the reported literature.

The experimental results obtained by the real flexural test and the results obtained by the simulated flexural tests of CFRP composites show a similar trend. The highest percentage difference of 14.25% between the simulation and the real experiment was found for the software tool Digimat FE–GG 630 T composite. The lowest difference of 0.5% was found for the software tool Digimat MF–GG 204 T composite. By comparing the results of the software tools with the results of the experimental measurements, it was found that the Digimat MF tool is closer to the results of the experimental measurements than the Digimat FE tool.

The results of limited real flexural tests and virtual simulations using MSC Marc Mentat 2022.3 and Digimat 2022.4 software show satisfactory agreement. By comparing the results of simulations using the Digimat MF and FE tools, it was found that the MF tool is more suitable for this type of material model from used composite materials. It is likely that the accuracy of the Digimat MF tool is satisfactory for reducing the number of real experimental specimens and thus reducing the amount of waste. Since only samples with an orientation of the longer side of the fabric (weft direction) were used in the tests, simulations and limited real experimental measurements of composite materials could be supplemented with other possible combinations of multi-layer and differently oriented layers. When determining the input parameters, it would be appropriate to determine the average value of the width and thickness of the yarn. The potential of used modern software showed that it is sufficient for carry out simulations and limited real tests with complex prototype models. Expensive, extensive and non-ecological testing of input materials need not be carried out.

The future research direction could lead to a more detailed examination of the input mechanical properties in material models and their comparison with other fibers, not only with carbon, similar to Wolter et al. [33]. The overall performance of the composites under high-load or high-temperature environments, as well as the impact of the negative coefficient of longitudinal thermal expansion of the carbon fibers when the composites are subjected to fluctuating thermal environments, can be studied in the future. An examination of microcracks, the inhomogeneity of the material, and the wear and abrasion resistance of composites can also be undertaken.

## Figures and Tables

**Table 1:** Properties of raw input materials.

Type of Material		Areal Weight [g/sqm]	Weaving Style	Material/Linear Density	Thickness [mm]
Prepreg	GG 204 T	204	Twill 2/2	3 K Carbon 200 tex	0.20
	GG 630 T	630	Twill 2/2	12 K Carbon 800 tex	0.62
Fabric	GG 285 T	285	Twill 2/2	3 K Carbon 200 tex	0.28
	GG 300 T	300	Twill 2/2	6 K Carbon 400 tex	0.29

**Table 2:** Properties of epoxy resin LG 120 and hardener HG 700.

Type of Material	Viscosity at 25 °C [g/cm^3^]	Density at 25 °C [mPa.s]
Resin LG 120	900–1200	1.18–1.23
Hardener HG 700	10–30	0.94

**Table 3:** Mechanical properties of cured unreinforced epoxy-LG 120 + HG 700.

Properties	Value	Units
Bending strength limit	110–120	MPa
Flexural modulus E	2900–3300	MPa
Ultimate tensile strength	75–85	MPa
Compressive strength limit	130–150	MPa
Ductility	5–6.5	%
Sudden muscularity	38–48	KJ/m^−2^

**Table 4:** Thermal properties of cured unreinforced epoxy-LG 120 + HG 700.

Curing at Temperature	Heat Resistance	Units
at 23 °C (2–7 days)	60	°C
at 50 °C (4 h)	70	°C
at 60 °C (>4 h)	80	°C
at 90 °C (>2 h)	115	°C
at 120 °C (2 h)	130	°C

**Table 5:** Specimen dimension after grinding.

Type of Material	Specimen	Width b [mm]	Thickness h [mm]
GG 204 T	1	14.75	1.97
	2	15.00	2.02
	3	14.99	1.98
GG 630 T	1	14.80	1.95
	2	15.00	1.96
	3	15.03	1.95
GG 285 T	1	15.00	2.61
	2	14.99	2.59
	3	15.05	2.62
GG 300 T	1	14.83	2.58
	2	14.80	2.55
	3	14.90	2.58

**Table 6:** Input data, yarn properties.

Type of Material	Filament Count	Fiber Dia. [mm]	Yarn Cross Section, Height [mm]	Yarn Cross Section, Width [mm]
GG 204 T	3 K	0.007	0.109	0.896
GG 630 T	12 K	0.007	0.22	1.61
GG 285 T	3 K	0.007	0.29	0.83
GG 300 T	6 K	0.007	0.32	0.67

**Table 7:** Average values of virtual specimen dimensions and average values of loading force F.

Material	Average Value Width b [mm]	Average Value Thickness h [mm]	Average Value Force F [N]
GG 204 T	14.9	1.99	142
GG 630 T	14.94	1.95	156
GG 285 T	15.01	2.6	445
GG 300 T	14.84	2.57	467

**Table 8:** Results of flexural test and value of flexural modulus of elasticity.

Material	Specimen nr.	Flexural Modulus E_f_ [GPa]	Average Flexural Modulus E_f_ [GPa]
GG 285 T	1	52.49	
	2	51.85	52.2 ± 0.33
	3	52.31	
GG 300 T	1	57.48	
	2	59.39	58.4 ± 0.96
	3	58.34	
GG 204 T	1	38.17	
	2	36.36	37.4 ± 0.94
	3	37.68	
GG 630 T	1	40.24	
	2	40.37	40.7 ± 0.69
	3	41.49	

**Table 9:** The results of flexural test of FEM simulation-deflection.

Material PROPERTY	GG 204 T MF	GG 204 T FE	GG 630 T MF	GG 630 T FE	GG 285 T MF	GG 285 T FE	GG 300 T MF	GG 300 T FE
Force [N]	142	142	156	156	445	445	467	467
Max. experimental deflection [mm]	4	4	4	4	4	4	4	4
Max. simulation deflection [mm]	3.98	3.69	3.77	3.85	4.13	4.57	4.29	4.53
Difference of deflections [mm]	0.02	0.31	0.23	0.15	1.53	1.84	0.29	0.53
Percentage difference [%]	0.5	7.75	5.75	3.75	3.25	14.25	7.25	13.25

**Table 10:** The results of flexural test of FEM simulation–flexural modulus.

Method	Material PROPERTY	GG 204 T	GG 630 T	GG 285 T	GG 300 T
Flexural test	Flexural modulus E_f_—[GPa]	37.4 ± 0.9	40.7 ± 0.7	52.2 ± 0.3	58.4 ± 1
Simulation MF	Flexural modulus E_11_, E_22_—[GPa]	36.34	41.657	50.981	51.008
	Flexural modulus E_33_—[GPa]	6.36	7.43	17.412	22.348
	Shear modulus G_12_—[GPa]	2.467	2.986	5.007	5.907
	Shear modulus G_31_, G_23_—[GPa]	2.165	2.887	5.414	6.134
	Poisson’s ratio μ_12_—[GPa]	0.0484	0.0437	0.0669	0.085
	Poisson’s ratio μ_23_—[GPa]	0.4078	0.443	0.32367	0.279
	Poisson’s ratio μ_31_—[GPa]	0.0714	0.079	0.11054	0.122
Simulation FE	Flexural modulus E_11_, E_22_—[GPa]	59.333	40.883	39.69	38.617
	Flexural modulus E_33_—[GPa]	9.426	6.542	8.58	9.645
	Shear modulus G_12_—[GPa]	4.64	3.747	3.65	4.18
	Shear modulus G_31_, G_23_—[GPa]	3.019	2.066	3.24	3.788
	Poisson’s ratio μ_12_—[GPa]	0.1048	0.126	0.163	0.176
	Poisson’s ratio μ_23_—[GPa]	0.386	0.392	0.438	0.44
	Poisson’s ratio μ_31_—[GPa]	0.0613	0.0628	0.095	0.11

## Data Availability

Data are contained within the article.
